# Do We Need a Cool-Down After Exercise? A Narrative Review of the Psychophysiological Effects and the Effects on Performance, Injuries and the Long-Term Adaptive Response

**DOI:** 10.1007/s40279-018-0916-2

**Published:** 2018-04-16

**Authors:** Bas Van Hooren, Jonathan M. Peake

**Affiliations:** 1 Department of Nutrition and Movement Sciences, Maastricht University Medical Centre+, NUTRIM School of Nutrition and Translational Research in Metabolism, Universiteitssingel 50, 6229 ER Maastricht, The Netherlands; 2 Institute of Sport Studies, Fontys University of Applied Sciences, Eindhoven, The Netherlands; 3 School of Biomedical Sciences and Institute of Health and Biomedical Innovation, Queensland University of Technology, Brisbane, Australia; 4 Sport Performance Innovation and Knowledge Excellence, Queensland Academy of Sport, Brisbane, Australia

## Abstract

It is widely believed that an active cool-down is more effective for promoting post-exercise recovery than a passive cool-down involving no activity. However, research on this topic has never been synthesized and it therefore remains largely unknown whether this belief is correct. This review compares the effects of various types of active cool-downs with passive cool-downs on sports performance, injuries, long-term adaptive responses, and psychophysiological markers of post-exercise recovery. An active cool-down is largely ineffective with respect to enhancing same-day and next-day(s) sports performance, but some beneficial effects on next-day(s) performance have been reported. Active cool-downs do not appear to prevent injuries, and preliminary evidence suggests that performing an active cool-down on a regular basis does not attenuate the long-term adaptive response. Active cool-downs accelerate recovery of lactate in blood, but not necessarily in muscle tissue. Performing active cool-downs may partially prevent immune system depression and promote faster recovery of the cardiovascular and respiratory systems. However, it is unknown whether this reduces the likelihood of post-exercise illnesses, syncope, and cardiovascular complications. Most evidence indicates that active cool-downs do not significantly reduce muscle soreness, or improve the recovery of indirect markers of muscle damage, neuromuscular contractile properties, musculotendinous stiffness, range of motion, systemic hormonal concentrations, or measures of psychological recovery. It can also interfere with muscle glycogen resynthesis. In summary, based on the empirical evidence currently available, active cool-downs are largely ineffective for improving most psychophysiological markers of post-exercise recovery, but may nevertheless offer some benefits compared with a passive cool-down.

## Key Points

**Table:** 

Many individuals regularly perform 5–15 min of low- to moderate-intensity exercises within approximately 1 h after their practice and competition (i.e., active cool-downs) in an attempt to facilitate recovery.
An active cool-down is largely ineffective at improving sports performance later during the same day when the time between successive training sessions or competitions is > 4 h. It is most likely ineffective at improving sports performance during the next day(s), but some beneficial effects have been observed.
An active cool-down does likely not attenuate the long-term adaptive response or prevent injuries.

## Introduction

**Fig. 1:**
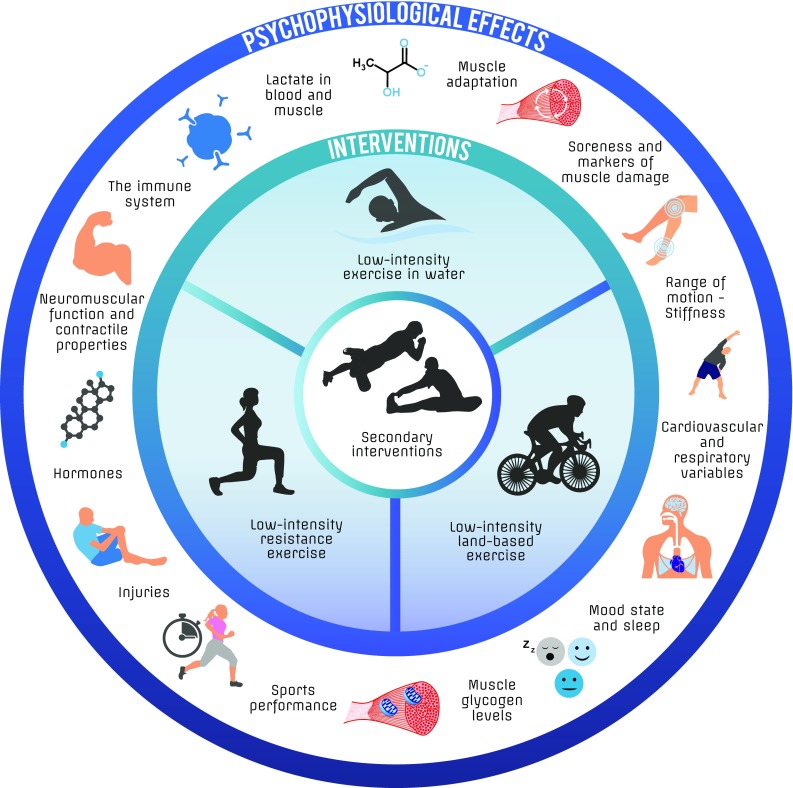
Infographic of active cool-down interventions and their commonly proposed psychophysiological effects

It is widely assumed that promoting physiological and psychological recovery after exercise allows individuals to perform better during subsequent training sessions or competition, and lowers the risk of injuries. Various recovery interventions are therefore used to facilitate recovery after exercise. The best known and most widely used post-exercise recovery intervention is (arguably) the active cool-down, which is also known as an active recovery or warm-down. Several surveys show that many team sport players and athletes participating in individual sports regularly perform 5–15 min of low- to moderate-intensity exercises within approximately 1 h after their practice and competition to facilitate recovery [1–8]. For example, a recent survey among collegiate athletic trainers in the USA found that 89% of the trainers recommended a cool-down, with 53% of these trainers recommending jogging as the preferred active cool-down method [1]. There is currently no formal definition of an active cool-down; here, we define it as an activity that involves voluntary, low- to moderate-intensity exercise or movement performed within 1 h after training and competition. Examples of active cool-down interventions and their suggested effects are shown in Fig. 1. The effects of recovery interventions such as cold-water immersion [9, 10], compression garments [11, 12], and cryotherapy [13, 14] have been reviewed extensively. By contrast, the active cool-down has never been thoroughly reviewed. It remains largely unknown whether an active cool-down offers any benefits compared with a passive cool-down (i.e., no cool-down), and thus whether it is an appropriate or effective recovery intervention.

The primary aim of this review is to synthesize the evidence as to whether an active cool-down enhances sports performance more effectively than a passive cool-down when performance is measured after approximately > 4 h after the initial exercise. This review also compares the physiological and psychological effects of an active cool-down to a passive cool-down, and discusses the effects of an active cool-down on injuries and the long-term adaptive responses to exercise training. The value of static stretching and foam rolling as cool-down interventions is briefly discussed in separate sections because these interventions are both frequently performed in combination with an active cool-down.

## Methods

There are various passive cool-down interventions such as sitting rest, saunas, pneumatic leg compression, and electrostimulation (see Table 1 for an overview) [15–23]. However, most non-elite athletes do not have access to a sauna or equipment for the other interventions, and most practitioners also lack the necessary knowledge about how best to apply these interventions (partly because of a lack of evidence-based guidelines). Even elite team sport players do not always have access to these recovery interventions when they play away games [24]. In the current review, we have therefore only included studies that have compared an active cool-down with a passive cool-down that consists of sitting, lying, or standing (without walking). Active cool-downs that combine exercise with cold water immersion [25] are also excluded. We have also restricted the review to studies that have investigated the effects of performing an active cool-down within approximately 1 h after exercise, because findings from a recent survey suggest that this most closely replicates the cool-down procedure of many recreational and professional athletes [7]. Studies that have applied an active recovery for several days after exercise are only discussed if they have (1) applied the active recovery within 1 h after exercise (i.e., active cool-down) and (2) evaluated recovery before applying the active recovery on the next day. Finally, we primarily focus on how active cool-downs influence performance and psychophysiological variables during successive exercise sessions or competitions [i.e., approximately > 4 h after exercise, or during the next day(s)]. This type of recovery has also been referred to as ‘training recovery’ [26]. Studies that have investigated the effects of active recovery between bouts of exercise with relatively short rest periods (e.g., 20 min) are excluded from the review. As such, the findings of this review will be of primary interest to athletes and practitioners who regularly use an active cool-down to facilitate recovery between training sessions or competitions, but are interested in what evidence exists that supports the use of an active cool-down compared with a passive cool-down. Relevant studies have been searched in the electronic databases of Google Scholar and Pubmed using combinations of keywords and Booleans that included (cool-down OR active recovery OR warm-down) AND (sports performance OR recover OR recovery OR physiological OR physiology OR psychological OR psychology OR injury OR injuries OR long-term adaptive response OR adaptation). Forward citation and reference lists of relevant articles were examined, and databases with e-published ahead of print articles from relevant journals were searched to identify additional articles.

**Table 1:** Overview of passive cool-down/recovery interventions

Sitting, standing, or lying rest	Cold-water immersion
Sauna	Hot-water immersion
Massage	Contrast-water therapy
Pneumatic leg compression	Cryotherapy
Peristaltic pulse dynamic compression	Crycompression therapy
External counterpulsation therapy	Flotation Restricted Environmental Stimulation
Compression garments	Hyperbaric oxygen therapy
Intermittent negative pressure	Foam rolling^a^
Vascular occlusion	Static stretching^a^
Local or whole-body vibration therapy	Neuromuscular electrical stimulation
Ultrasound therapy	Sustained heat treatment
Photo-/light-emitting diodes therapy	

Passive recovery interventions are defined here as involving no or minimum voluntary/intentional exercise or movement

^a^These passive recovery interventions are frequently used in combination with active cool-downs

## Effects on Sports Performance

In principle, better psychophysiological recovery following exercise may attenuate or prevent performance decrements—or even enhance performance—during a subsequent training session or competition [27]. The following sections discuss the effects of an active cool-down on measures of physical performance such as vertical jump height and sprint performance measured later during the same day or during the next day(s).

### Same-Day Performance

Elite athletes often train or compete more than once a day, so recovery interventions between training sessions or events may help to restore exercise performance. This section only discusses studies that have investigated the effects of an active cool-down after at least 4 h of rest between training sessions or competitions to reflect the effects of an active cool-down on ‘training recovery’ [26].

Relatively few studies have investigated the benefits of active cool-downs on performance measured > 4 h after exercise, and these studies generally found trivial (statistically non-significant effects), and sometimes even small (non-significant) detrimental effects of an active cool-down on performance [15, 28–30] (Table 2). For example, Tessitore et al. [28] compared a 20-min active cool-down (consisting of either land-based or water-based aerobic exercises and stretching) with a passive cool-down following a standardized soccer training in elite youth players. After a 4-h rest period, the athletes performed several anaerobic performance tests. Both active cool-down protocols had trivial to small (negative) non-significant effects on anaerobic performance, such as 10-m sprint time and vertical jump height. In a later study on futsal players, similar cool-down interventions also had trivial to small (negative) non-significant effects anaerobic sports performance measured 4.5 h after a friendly match compared with a passive cool-down [29]. Therefore, whereas active recovery generally does benefit sports performance when the time between successive performances is short (10–20 min) [31–35], the findings from the studies above indicate overall that an active cool-down does not improve sports performance later on the same day when time between successive performances is > 4 h and may even have small detrimental effects. However, more research on the effects of active cool-downs following others forms of exercise is needed.

**Table 2:** The effects of active cool-downs on same-day and next-day performance

Study	Participants (mean age ± standard deviation)	Fatiguing exercise	Active cool-down duration, modality, and intensity	Interval between end cool-down and subsequent performance (h)	Outcome measures	Results (% difference; ± 90% CIs for between-group comparison [when available], qualitative description of the probability and effect magnitude)*
*Same day performance*						
Cortis et al. [15]	8 military men (21.9 ± 1.3 years)	Incremental running test	16 min shallow water-aerobic exercises at 60% HR_max_ and 4 min stretching	4.5	CMJ	Pre-afternoon training: 0.0%, trivial Post-afternoon training: 0.0%, trivial
					BJ	Pre-afternoon training: − 4.0%, small Post-afternoon training: − 7.8%, small
					*V*O_2_ at various running velocities	6 km/h: − 5.1%, small 8 km/h: 4.7%, small 10 km/h: − 3.1%, small 12 km/h: − 5.6%, small
Tessitore et al. [28]	12 young professional male soccer players (18.1 ± 1.2 years)	100 min standardized soccer training	16 min low-intensity dry-aerobic exercises and 4 min stretching or 16 min shallow water exercises and 4 min stretching	4	SJ	Dry: − 1.2%, trivial Water: 1.5%, trivial
					CMJ	Dry: − 1.7%, small Water: 2.9%, small
					BJ	Dry: 0.0%, trivial Water: − 4.2%, small
					10-m sprint	Dry: − 3.7%, moderate Water: 0.0%, trivial
Tessitore et al. [29]	10 male futsal players (23 ± 2 years)	1 h futsal game	16 min low-intensity dry-aerobic exercises and 4 min stretching or 16 min shallow water exercises and 4 min stretching	4.5	CMJ	Dry: − 2.8%, small Water: − 4.6%, small
					BJ	Dry: − 3.7%, small Water: − 1.7%, trivial
					10-m sprint	Dry: 0.0%, trivial Water: − 1.1%, trivial
Reader et al. [30]	8 male and 1 female elite weightlifters (26.5 ± 4.8 years)	Olympic weightlifting exercises and various derivatives such as back squat and push press	15 min supervised rowing ergometer at 1 W/kg body weight and stroke frequency of < 20/min	4.25	CMJ	Session 1–2: − 4.6; ± 3.2%, *likely* small Session 3–4: 1.7; ± 3.9%, *unclear, possibly* trivial
*Next day performance*						
Vanderthommen et al. [36]	19 healthy men (23.4 ± 2.1 years)	3 × 25 isometric contractions of the knee extensors at 60 55 and 50% of MVC	25 min pedaling on stationary bicycle at 60 rpm (approx. 50% HR_max_)	24	MVC	4.7; ± 8.0%, *unclear*, *possibly* small
Weber et al. [37]	40 untrained females (22.9 ± 3.7 years)	Eccentric arm-curls until fatigue	8 min upper body ergometry at 60 rpm	24	MVIC	1.5%, trivial
					Peak torque at 60˚/s	− 7.5%, small
Rey et al. [38]	31 professional male soccer players (23.5 ± 3.4 years)	45 min standardized soccer training	20 min low-intensity exercises (12 min running at 65% maximum aerobic velocity and 8 min stretching)	24	CMJ	6.6; ± 5.3%, *unclear*, *likely* moderate
					20-m sprint	− 0.6; ± 3.5%, *unclear*, *possibly* trivial
					Balsom agility test	− 0.7; ± 0.7%, *likely* trivial
Lane and Wenger [39]	10 physically active men (26.3 ± 6.3 years)	18-min intermittent cycling protocol	15 min cycling at 30% *V*O_2max_	24	Work completed during a cycling protocol	1.7%, trivial
Takahashi et al. [40]	10 male long-distance runners (20 ± 1 years)	3 sets of 5-min downhill treadmill running at a speed corresponding to their individual best 5000 m time	30 min of aqua exercises (walking, jogging, jumping)	24	Muscle power of leg extensors in leg press	15; ± 12%, *unclear likely* moderate
					Whole-body reaction time	− 2.4%, trivial
Dawson et al. [41]	17 Western Australian Football League (WAFL) players (24.2 ± 2.9 years)	Football matches	15 min of pool walking	14	6-s cycle sprint peak power	3.2; ± 2.7%, *likely* small
					6-s cycle sprint time to peak power	− 2.7%, small
					6-s cycle sprint total work	3%, small
					CMJ	8.1; ± 6.7%, *unclear likely* moderate
King and Duffield [42]	10 trained female netball players (19.5 ± 1.5 years)	4 × 15 min intermittent-sprint exercise circuit	15 min low-intensity exercise at 40% of maximum aerobic speed	24	5 CMJs in 20 s	Pre-exercise: − 25%, small Post-exercise: − 29%, small
					5 20-m sprints	Pre-exercise: 62%, moderate Post exercise: − 6.1%, trivial
Wahl et al. [43]	20 male sport students (24.4 ± 2.2 years)	300 × maximal effort CMJs	30 min aqua biking at 65-75 rpm	24, 48, and 72	MVIC	24 h: 4.0%^†^, small 48 h: 2.2%^†^, trivial 72 h: 3.1%^†^, small
					Repetitions with 30% MVIC	24 h: 4.7%^†^, trivial 48 h: 14%^†^, small 72 h: 11%^†^, trivial
Getto and Golden [44]	23 (13 male) and 10 female) Division I collegiate athletes (age not reported)	Conditioning session that included sprinting, plyometrics and change of directions	2 sets of 30 s forward walking with variations on walking on underwater treadmill at 1.0–1.5 mph	24–28	CMJ	0.2%, trivial
					20-m sprint	− 18%, moderate
Marquet et al. [45]	11 world-class elite BMX riders (7 male, 4 female; 20.9 ± 2.1 years)	High-intensity interval training and maximum intensity resistance training	Pedaling at 70% *V*O_2max_ for 2 × 5 min separated by 5 min passive recovery	Next day, but hours are not reported	Maximum power	Pre-training: 0.2%, trivial Post-training: 1.7%, trivial
					Maximum cadence	Pre-training: − 2.1%, trivial Post-training: − 0.8; ± 0.6%, *most likely* trivial
Taipale et al. [46]	18 physically active men (25.6 ± 3.5 years)	Bilateral leg press with 10 × 10 reps at 70% of 1RM	Bilateral leg press with 10 × 10 at 30% 1RM with 5 min passive rest between sets	18	CMJ	33%, moderate
					MVIC	9.7%, trivial
Reilly and Rigby [47]	14 male students (soccer players; 20.9 ± 1.5 years)	Soccer match	5 min jogging, 5 min stretching, 5 min leg ‘shake down’ by other player	24 and 48	Broad jump	Significant improvement by 9 cm in active cool-down compared to deterioration by 7 cm in passive cool-down at 24 h. Difference remained significant at 48 h
					Vertical jump	Significant improvement by 2.5 cm in active cool-down compared to deterioration by 1 cm in passive cool-down at 24 h. Difference remained significant at 48 h
					3 30-m sprints	0.22 s (5%) slower in passive cool-down group at 24 h and 0.6 s at 48 h
					Sprint-fatigue test (7 30-m sprints with 20 s rest)	At 48 h, mean performance was not significantly different from baseline in active cool-down group
Crowther et al. [25]	34 recreationally active males (27 ± 6 years)	3 × 15 min simulated team-game circuit	14 min jogging at 35% of peak speed obtained during maximum sprints^††^	24 and 48	Time on repeated-sprint test	24 h: 0.4; ± 1.4%, *unclear, possibly* trivial 48 h: − 0.9; ± 1.8%, *possibly* trivial
					CMJ relative peak power (best jump)	24 h: − 1.9; ± 1.6%, *likely* trivial 48 h: − 0.6; ± 1.4%, *very likely* trivial
					CMJ relative peak power (average of jumps)	24 h: − 2.2; ± 1.7%, *possibly* trivial 48 h: − 1.2; ± 1.6%, *likely* trivial
Reader et al. [30]	8 male and 1 female elite weightlifters (26.5 ± 4.8 years)	Olympic weightlifting exercises and various derivatives such as back squat and push press	15 min supervised rowing ergometer at 1 W/kg body weight and stroke frequency of < 20/min	16	CMJ	Session 2–3: − 0.32; ± 4.4%, *likely* trivial Session 4-after: 0.92; ± 3.5%, *possibly* trivial

*HR*_*max*_ maximum heart rate, *CMJ* countermovement jump, *SJ* squat jump, *BJ* bounce jump, *MVIC* maximum voluntary isometric contraction, *V*O_2*ma*x_ maximum oxygen uptake, *RM* repetition maximum

*Percentage differences were calculated by first computing a factor difference within the active and passive cool-down group by dividing the post cool-down mean (e.g., > 4 h same-day or next-day performance) by the post fatiguing exercise, but pre-cool-down mean. When no post fatiguing exercise, but pre-cool-down mean was reported, the pre-fatiguing exercise mean was used to calculate the within group factor difference. The factor of the active cool-down group was then divided by the factor difference of the passive cool-down group and converted to a percentage effect, whereby negative and positive values reflect worse and better performance of the active cool-down group, respectively. When an exact p-value or *p* < 0.05 was reported, a statistical spreadsheet [48] was used to derive 90% confidence intervals of the percentage difference. Standardizes differences were calculated by first computing a standardized difference within the active and passive cool-down group and then subtracting the passive cool-down standardized difference from the active cool-down standardized difference. The standardized difference for each group was calculated by subtracting the post fatiguing exercise, but pre-cool-down mean from the post cool-down mean divided by the pre-cool-down pooled standard deviation from both groups. The standardized difference was corrected for small sample size bias (i.e., Hedges’s *g*_*s*_) as outlined by Lakens [49]. When no post fatiguing exercise, but pre-cool-down mean was reported, the pre-fatiguing exercise mean and standard deviation were used to calculate the standardized difference. Standardized differences were expressed qualitatively using the following scale: < 0.2, trivial; 0.2–0.6, small; 0.6–1.2, moderate; 1.2–2.0 large; > 2.0, very large [50]. When an exact *p* value or *p* < 0.05 was reported, the probability that the (true) difference in performance was better (beneficial), similar (trivial) or worse (harmful) in relation to the smallest worthwhile change (0.2 multiplied by the pooled between-subject SD for measures of team sports performance and indirect measures of solo sports performance) was calculated using a statistical spreadsheet [48]. Quantitative probabilities of beneficial, similar or worse performance were assessed and reported qualitatively using the following scale: 25–75%, *possibly*; 75–95%, *likely*; 95–99.5, *very likely*; > 99.5%, *most likely*. If the probability of benefit was > 25%, but the probability of harm was > 0.5%, the true differences were considered *unclear* (i.e., clinical magnitude-based inference). In this case, the largest probability for a change was reported to give an indication of the most likely change [50]. When insufficient data were reported for any of these calculations, these data were requested from the corresponding authors by e-mail

^†^Standardized differences are estimated based on the results reported in Fig. 3 in reference [43]

^††^The passive cool-down group also performed 5 min of jogging prior to the passive cool-down

### Next-Day(s) Performance

Conflicting findings have been reported with regard to the effects of an active cool-down on next-day(s) performance, with some studies reporting small to moderate magnitude benefits of an active cool-down compared with a passive cool-down, and others reporting trivial effects or small decreases (Table 2) [25, 30, 39–49]. Most studies, however, report trivial effects, with some studies reporting beneficial effects and only a few studies reporting harmful effects. For example, a study on sport students found that an aqua cycling active cool-down had small to trivial effects on recovery of maximum voluntary isometric contraction (MVIC) force and muscular endurance at 24, 48, or 72 h post-exercise compared with a passive cool-down [45]. In contrast, in a group of female netball players, a 15-min active cool-down consisting of low-intensity running resulted in a moderate magnitude decrease of 20-m sprint time and a small decrease in vertical jump height 24 h after a simulated netball game compared with a passive cool-down [44]. Interestingly, a study on well-trained long-distance runners found that muscle power (as measured during a leg press movement) was likely higher 1 day after downhill running in the group that performed a water-based active cool-down compared with the group that performed a passive cool-down, while whole-body reaction time showed a small decrease [40]. Finally, a study on professional soccer players found that an active cool-down had a likely beneficial effect on countermovement jump performance 24 h after a standardized training session, while 20-m sprint and agility performance showed small harmful and trivial effects, respectively [50]. Overall, these conflicting findings may be related to the type of cool-down performed, the exercise that precedes the cool-down, the training experience of the individuals and the individual preferences and believes. It should be noted that all studies investigated high-intensity performances such as jumping and sprinting and more research is required on endurance performance.

## Physiological Effects of an Active Cool-Down

An active cool-down is believed to have many physiological benefits compared with a passive cool-down, such as a faster recovery of heart rate, less muscle soreness, and more rapid reduction of metabolic by-products [7]. The evidence for these supposed physiological benefits is reviewed in the following sections.

### Removal of Metabolic By-Products

High-intensity exercise can lead to an accumulation of metabolic by-products in muscle such as lactate, which has traditionally been associated with fatigue [51]. As a result, the rate at which the lactate concentration is reduced in blood—and to a lesser extent, muscle tissue—has frequently been used as an objective indicator of recovery from exercise. A large body of research has shown that a variety of low- to moderate-intensity active cool-down protocols are more effective than a passive cool-down for removing lactate from blood [52–69] and muscle tissue [58, 64]. However, there are some conflicting findings, with some studies reporting no significant difference—and sometimes even a slower removal of lactate in blood [44, 70] or muscle [66, 68]—as a result of an active cool-down. Regardless, the functional benefit of faster lactate removal is debatable. For example, several studies found no significant difference between an active cool-down and a passive cool-down in the blood lactate concentration measured more than 20 min after exercise [45, 67]. Blood lactate returns to resting levels after high-intensity exercise within approximately 20–120 min—even without any post-exercise activity [55, 60, 71]. Even elite athletes do not usually perform another training session within 90 min after the preceding session; faster removal of lactate by an active cool-down may therefore be largely irrelevant [72]. A decrease in blood lactate concentration may also not be an appropriate indicator of recovery following exercise [51, 72]. Among those studies that have reported a faster removal of blood lactate following an active cool-down, subsequent exercise performance was not always improved [67, 72].

Although it has traditionally been assumed that lactic acid production results in metabolic acidosis, it has been argued that lactate production coincides with cellular acidosis, but is not a direct cause of and even retards metabolic acidosis [73]. It is therefore important to consider the potential differential effects of an active cool-down on blood or muscle lactate removal and metabolic acidosis. An active cool-down results in a faster return of blood plasma pH and intramuscular pH to resting levels [64, 74]. This effect may preserve neuromuscular function by reducing the effects of exercise-induced acidosis, which affects the functioning of glycolytic enzymes such as phosphorylase and phosphofructokinase. However, one study investigated the effects of an active and passive cool-down on pH levels up to 16 min after exercise [74], whereas the other study investigated pH levels until 80 min after exercise [64]. This latter study found no significant effect of an active cool-down on blood pH levels 80 min after exercise. The relevance of these findings for improved performance during a training session or competition later on the same day (i.e., > 4 h) or the next day(s) is therefore questionable.

In summary, compared with a passive cool-down, an active cool-down generally leads to a faster removal of blood lactate when the intensity of the exercise is low to moderate. However, the practical relevance of this effect is questionable. Lactate is not necessarily removed more rapidly from muscle tissue with an active cool-down. Finally, an active cool-down leads to a faster recovery of pH to resting levels.

### Delayed-Onset Muscle Soreness

An active cool-down increases the blood flow to muscles and skin [58, 75] (see Sect. 4.8). This increase in blood flow may reduce the accumulation of metabolic by-products and factors associated with muscle soreness (e.g., cyclo-oxygenase and glial cell line-derived neurotrophic factor [76]) and accelerate muscle repair and remodeling. Several studies have investigated whether an active cool-down does indeed attenuate delayed-onset muscle soreness. It should be noted, though, that some studies [40, 45, 48, 77, 78] used exercise protocols that induce severe delayed-onset muscle soreness, but are seldom used in everyday athletic training. Therefore, the findings of these studies do not necessarily apply to ‘normal’ training sessions that induce less delayed-onset muscle soreness.

Most studies among both recreationally active individuals and professional athletes have found no significant effect of an active cool-down on delayed-onset muscle soreness or tenderness at different times following exercise (i.e., ranging from immediately after exercise up to 96 h after exercise) compared with a passive cool-down [15, 25, 29, 40, 41, 45, 46, 48, 49, 77–80]. For example, Law and Herbert [77] compared the effects of an active cool-down consisting of uphill walking versus a passive cool-down on delayed-onset muscle soreness in healthy adults following backwards downhill walking on an incline treadmill (to induce muscle damage). The active cool-down did not significantly reduce delayed-onset muscle soreness or tenderness at 10 min, 24, 48 or 72 h following exercise. Interestingly, a study on netball players found that an active cool-down consisting of low-intensity running after a simulated netball match actually resulted in greater muscle soreness immediately after the active cool-down compared with a passive cool-down, but there was no significant difference 24 h after the match [44]. The running cool-down itself may have caused extra muscle damage, resulting in the higher rating of muscle soreness immediately after the cool-down. Higher impact weight-bearing cool-down activities such as running may therefore exacerbate delayed-onset muscle soreness immediately after exercise, but more research is required to substantiate this notion.

In contrast with the studies above, another study involving young professional soccer players reported that the mean subjective rating of muscle soreness was significantly lower 4–5 h after an active cool-down consisting of low-intensity exercises such as jogging compared with a passive cool-down [28]. Interestingly, there was no significant difference in muscle soreness compared with a passive cool-down when these same exercises were performed in water, suggesting that any hydrostatic effects of water immersion did not reduce muscle soreness. Similarly, a study on world-class BMX riders found that an active cool-down consisting of 2 × 5 min of cycling at 70% of the maximum aerobic power reduced muscle soreness during the next day when compared with a passive cool-down [47]. It could be argued that these conflicting findings are related to differences in the physical fitness of the individuals. For example, the netball players were not as highly trained as the soccer players and BMX riders. For non-elite athletes, an active cool-down therefore generally has no effect on delayed-onset muscle soreness, whereas it may have a beneficial effect for better trained individuals. However, other studies among well-trained individuals have also reported no beneficial effects of active cool-down on delayed-onset muscle soreness [29, 41, 80], while a study among student soccer players reported beneficial effects of an active cool-down combined with stretching and a ‘leg shake down’ on muscle soreness [42]. These findings suggest that other factors such as the intensity and duration of the exercise and cool-down, and the timing of soreness assessment may also influence the effectiveness. In summary, these findings indicate that an active cool-down is generally not effective for reducing delayed-onset muscle soreness following exercise.

### Indirect Markers of Muscle Damage

The perception of muscle soreness does not necessary reflect actual muscle damage [81, 82]. Therefore, even though an active cool-down is generally not effective for reducing delayed-onset muscle soreness, it may have beneficial effects on other markers of muscle damage.

Studies that have investigated the effects of an active cool-down on indirect markers of muscle damage from immediately after exercise up to 84 h after exercise have reported conflicting findings. Two studies observed significantly faster recovery of these markers as a result of an active cool-down [70, 83], whereas three other studies found no significant difference [40, 45, 84]. For example, Gill et al. [83] reported a significantly faster recovery of creatine kinase activity in interstitial fluid in elite rugby players between 1 and 4 days after a rugby match combined with a cycling-based active cool-down compared with a passive cool-down. By contrast, a study comparing an aqua-cycling active cool-down and a passive cool-down in sport students found no significant difference in serum creatine kinase and lactate dehydrogenase activity, or myoglobin concentrations at 4, 24, 48, or 72 h after exercise [45]. These conflicting findings may be related to differences in the severity of muscle damage induced by exercise, the individual markers of muscle damage, and the type of cool-down protocol. It should be noted that frequently used indirect markers of muscle damage (e.g., creatine kinase activity) may not accurately reflect actual muscle damage [85–88]. Malm et al. [85] suggested that serum creatine kinase activity is more related to muscle adaptation than to muscle damage. Therefore, it is debatable whether a faster recovery of these indirect markers accurately reflects enhanced recovery.

Measures of strength and power are also frequently used as indirect markers of muscle damage. A study on untrained females found no significant effect of an active cool-down consisting of upper body ergometry on the recovery of the MVIC and peak torque 24 h after eccentric exercise of the elbow flexors [48]. Similar results were found in other studies on sport science students [45], physically active men [43], and healthy men [49]. However, most studies usually reported a slightly (non-significant) better recovery compared with the passive cool-down group (Table 2).

In summary, there are conflicting findings with regard to the effects of an active cool-down on indirect markers of muscle damage, with most studies reporting no significant beneficial effect of an active cool-down. Moreover, the relation of some of these markers with actual muscle damage is questionable—that is, a faster recovery of these markers does not necessarily correspond to a faster reduction in actual muscle damage.

### Neuromuscular Function and Contractile Properties

High-intensity exercise can induce central and peripheral fatigue, which may impair exercise performance during subsequent training or competition. Compared with a passive cool-down, Lattier et al. [89] did not find a significant effect of an active cool-down consisting of 20 min of running on the recovery of neuromuscular function (e.g., central activation, twitch mechanical, and M-wave characteristics) up to 65 min after high-intensity exercise. Similarly, a study on professional soccer players found no significant effect of an active cool-down consisting of combined low-intensity running and static stretching on muscular contractile properties such as biceps femoris contraction time and maximal radial displacement time (as measured by tensiomyography) 24 h after exercise [80]. Finally, an active cool-down consisting of aqua exercises also did not significantly affect whole-body reaction time, muscle contraction time or nerve reaction time in long-distance runners 24 h after exercise [40].

In summary, these findings indicate that an active cool-down does not significantly affect the recovery of neuromuscular function or contractile properties. However, in all studies there were generally small but non-significant positive effects of the active cool-down recovery on the recovery of neuromuscular function and contractile properties.

### Stiffness and Range of Motion

Damage to musculotendinous tissue as a result of exercise—specifically eccentric exercise—can increase the stiffness of the musculotendinous unit. This stiffness can persist for several days following exercise [90]. The increased passive musculotendinous stiffness can reduce the range of motion during subsequent training or competition [90], and this may impair performance. Researchers and trainers frequently use perceived flexibility and measures of flexibility such as the sit-and-reach test to assess recovery [91]. Another common belief for using an active cool-down is that it attenuates the decrease in range of motion [7] and increase in musculotendinous stiffness following exercise.

The scientific evidence available suggests that an active cool-down does not significantly attenuate the decrease in range of motion and perceived physical flexibility, or attenuate the increase in musculotendinous stiffness up to 72 h after exercise [25, 40, 41, 45, 50, 67, 92]. Takahashi et al. [40] found that an active cool-down consisting of 30 min of water exercises did not significantly affect sit-and-reach score, ankle range of motion, stride length, or calf and thigh musculotendinous stiffness measured 1 day after 3 × 5 min of downhill running. Similarly, a study among professional soccer players found no significant effect of an active cool-down consisting of 12 min submaximal running combined with 8 min of static stretching on lower limb flexibility 24 h after a standardized training program (consisting of 15 min of maximal intensity intermittent exercises and a 30 min of specific aerobic endurance drill) [50].

In summary, these findings indicate that an active cool-down does not attenuate the decrease in range of motion or the increase in musculotendinous stiffness following exercise.

### Muscle Glycogen Resynthesis

High-intensity exercise can deplete muscle glycogen storage, and this can impair subsequent high-intensity exercise performance up to 24 h post-exercise [93]. Strategies that enhance the resynthesis of glycogen may therefore attenuate the decrease in performance and even enhance performance. Athletes often consume carbohydrates after exercise. An active cool-down may theoretically enhance glycogen resynthesis, because an increased blood flow and elevated muscle temperature could increase glucose delivery to muscle tissue [94], while muscle contraction may increase the expression of the GLUT-4 glucose transporter. However, studies have found either no significant difference in the rate of glycogen resynthesis between an active cool-down and passive cool-down [58, 66, 95], or less glycogen resynthesis during an active cool-down [64, 68, 96–98]. During the active cool-down, these studies provided no carbohydrate [58, 64, 66, 68, 95], less carbohydrate [96], or more carbohydrate [97, 98] than what is recommended (1.2 g/kg/h [99]) for restoring muscle glycogen. Therefore, these findings suggest that an active cool-down may interfere with muscle glycogen resynthesis, particularly within type I muscle fibers [64], because these fibers are preferentially recruited during a low- to moderate-intensity active cool-down. Although this effect may be beneficial to enhance cellular responses and adaptation during a subsequent low- to moderate- intensity training (i.e., ‘train low’ [100]), it may also decrease performance during high-intensity training or competition. It should be noted that several studies applied active cool-downs for a duration that is rarely used in daily practice (e.g., 45 min up to 4 h) [64, 66, 96–98]. For example, Kuipers et al. compared glycogen resynthesis between a passive cool-down and an active cool-down in which participants cycled for 2.5 h at 40% of their maximum workload [97], or 3 h at 40% of their maximum workload [64, 66, 96, 98]. In contrast, studies that reported no significant (but also lower) difference in the rate of glycogen resynthesis between an active cool-down and passive cool-down usually applied shorter active cool-down durations (i.e., 10, 15, and 45 min [58, 66, 95]), suggesting that shorter active cool downs interfere less with glycogen resynthesis.

### Recovery of the Immune System

During the recovery period from high-intensity or prolonged exercise, there can be a temporary depression of the immune system (also referred to as an ‘open window’) during which microbial agents such as viruses have an increased chance to cause an infection or illness [101]. A faster recovery of the immune system following exercise can potentially reduce the chance of upper respiratory illnesses. A small number of studies have investigated the effects of an active cool-down on the recovery of the immune system up to 72 h after exercise.

Wigernaes et al. [70, 102] found that an active cool-down largely prevented the fall in white blood cell count immediately after exercise compared with a passive cool-down. However, there was no significant difference 120 min after the exercise [70]. Similarly, two other studies reported no significant difference between an active cool-down and passive cool-down on immune system markers 24 h after a soccer [103] and rugby match [84].

In summary, these findings suggest that an active cool-down may partially prevent the depression of circulating immune cell counts immediately after exercise, but this effect is probably negligible > 2 h after exercise. No studies have investigated the effects of regular active cool-downs, so it remains unknown whether this leads to fewer illnesses.

### Cardiovascular and Respiratory Variables

The cardiovascular and respiratory systems are highly active during exercise to supply the exercising muscles with blood and oxygen. These systems do not immediately return to resting levels after exercise, but remain activated for a considerable amount of time. For example, heart rate remains slightly elevated above resting heart rate for a relatively long time after exercise, with the exact period dependent on the intensity and duration of the exercise [104]. An active cool-down is frequently performed in an attempt to restore normal activity of these systems after exercise [7].

In a comparison between a passive cool-down and two cycling-based active cool-down protocols, Takahashi and Miyamoto [104] found that heart rate initially recovered in a nearly identical way, but 10 min after the exercise (3 min after the active cool-down), heart rate was significantly lower for the active cool-down interventions. A later study confirmed these findings, and suggested that this response to active cool-down reflected a faster restoration of vagal and sympathetic tone [105]. In one additional subject, it was shown that the heart rate following a passive cool-down was still higher 30 min after exercise than the resting heart rate, whereas it had returned to resting levels after the active cool-down [104]. By contrast, other studies found a slower heart rate recovery during an active cool-down compared with a passive cool-down. Nevertheless, these studies only monitored the heart rate for 60 s [106] or 5 min [107, 108] after exercise, and the practical relevance of these findings with regard to ‘training recovery’ is therefore limited.

An active cool-down has also been reported to lead to a faster recovery of respiratory variables such as minute expiratory ventilation, although this primarily occurred during the initial 20 s of the cool-down [109]. Other studies found a lower breathing frequency (non-significant) after an active cool-down [105] and a faster recovery of oxygen debt during an active cool-down [55].

Finally, the period right after exercise can be considered as a vulnerable period during which individuals can experience post-exercise syncope, with symptoms such as lightheadedness, tunnel vision, and blurred vision [110]. In severe circumstances, individuals may lose consciousness completely during this post-exercise period. It has been suggested that an active cool-down may prevent post-exercise syncope and cardiovascular complications by: (1) increasing blood flow to the heart and brain due to the contractions of the muscles [108, 110], (2) decreasing blood pooling in the lower extremities [104], and (3) theoretically preventing an increase in the partial pressure of arterial carbon dioxide [111]. Indeed, an active cool-down has been reported to result in a higher blood flow to the legs [58, 104] and forearm [75], but whether these effects prevent post-exercise syncope and cardiovascular complications remains unknown.

In summary, these findings suggest that an active cool-down may result in a faster recovery of the cardiovascular and respiratory system after exercise. However, it is unknown whether this also leads to a reduction in the incidence of post-exercise syncope and cardiovascular complications.

### Sweat Rate and Thermoregulation

Similar to the cardiovascular and respiratory systems, muscle and core temperature can remain elevated above resting levels up to 90 min after exercise. Sweat rate is higher after exercise to reduce the core temperature to resting levels [112]. Although an active cool-down on a stationary bike results in a higher sweat rate compared with a passive cool-down, core temperature is not lower even after 30 min of active cool-down [65, 75, 113–116]. Therefore, an active cool-down performed on a stationary bike does not result in a faster recovery of core temperature compared to a passive cool-down. Whether an active cool-down performed while moving (e.g., running outside during which sweat may evaporate faster compared with stationary biking) results in a faster recovery of core temperature compared with a passive cool-down requires further investigation.

### Hormone Concentrations

It has been proposed that the rate at which hormone concentrations return to resting levels can be used to characterize physiological stress [43] and psychological recovery [29]. The findings of four studies suggest that an active cool-down does not facilitate the recovery of hormone concentrations compared with a passive cool-down [29, 43, 64, 102]. A study on well-trained futsal players, for example, found no significant effect of an active cool-down on hormone concentrations measured 5 h after a futsal game or measured the next morning [29]. An active cool-down consisting of uphill treadmill running actually resulted in a slower acute restoration of plasma adrenaline, noradrenaline and cortisol concentrations compared with a passive cool-down [102]. However, from 30 min post-exercise onwards, there were no significant differences in the hormone concentrations. The relevance of this finding is therefore questionable. A later study reported similar findings, with the hormonal concentrations returning more slowly to resting levels compared with a passive cool-down, but there was no significant difference beyond 30 min post-exercise [64]. Finally, Taipale et al. [43] reported that an active cool-down consisting of 10 × 10 repetitions of leg press at 30% of the 1 repetition maximum did not result in significant between-group differences for several hormonal concentrations during the next morning.

In summary, these findings suggest that an active cool-down may result in a slower recovery of hormone concentrations immediately after exercise, but does not significantly affect the recovery of hormonal concentrations beyond 30 min post-exercise compared with a passive cool-down. In support of this, plasma concentrations for several hormones have been reported to return to resting levels within 60–120 min post-exercise even with a passive cool-down [117].

### Mood State, Self-Perception, and Sleep

Most research has investigated the physiological effects of an active cool-down and a passive cool-down, yet psychological effects are intimately linked to the physiological effects, and are also of major importance for performance. A recent systematic review even proposed that subjective measures of well-being better reflect training loads than do objective measures [118]. Therefore, the psychological effects of an active cool-down are also important to consider in relation to recovery.

Most studies have not reported any significant effect of an active cool-down on measures of psychological recovery such as the score on the Profile of Mood States (POMS) or rest-Q sport questionnaire. Nevertheless, the participants usually perceived an active cool-down as more beneficial than a passive cool-down [15, 25, 29, 30, 39, 41, 46, 47, 67, 119]. For example, a study among well-trained futsal players reported that the players perceived the active cool-down consisting of low-intensity exercises on land and especially the active cool-down consisting of water-based exercises as more beneficial than a passive cool-down—even though there was no significant effect on the recovery-stress state and the amount of sleep [29]. Another study among military men also did not demonstrate any significant effect of an active cool-down consisting of water exercises on sleep, rest-recovery score or rating of perceived exertion during submaximal exercise after a 6-h rest period [15]. However, the participants in this study did rate the water-based active cool-down as more beneficial than the passive cool-down. Interestingly, a study on sport students found no significant difference between a passive cool-down and an aqua-cycling active cool-down for perceived physical state 4, 24, 48, or 72 h after performing 300 countermovement jumps, but the perceived physical fitness and energy were slightly lower 24 h after the active cool-down [45]. Similarly, a study on recreational netball players reported that rating of perceived exertion was significantly higher following a 15-min running-based active cool-down compared with a passive cool-down [44]. These findings possibly reflect the greater energy expenditure associated with an active cool-down versus a passive cool-down. By contrast, a study among 15 rugby players found that the ‘tension’ score on the POMS questionnaire was significantly lower two days after a rugby match in the group that performed a 1-h active cool-down once a day compared with another group that performed a passive cool-down [84]. However, there was no significant effect on any of the other POMS scores, and no significant difference on the day after the match, when only one active cool-down session was performed. These findings imply that an active cool-down can potentially interfere with psychological recovery in untrained or recreationally trained individuals, whereas it likely has no (or a slight) positive effect on psychological recovery in better trained individuals. In support of this, even though most individuals perceive an active cool-down as more beneficial, some (recreationally active) individuals may perceive it as ‘more exercise’ or increasing stiffness [25]. This may explain why elite rugby players rated an active cool-down as more effective than amateur rugby players in a recent survey [6].

In summary, an active cool-down generally does not substantially influence measures of psychological recovery after exercise, but most individuals nevertheless perceive an active cool-down as more beneficial than a passive cool-down. Reasons reported for doing an active cool-down include relaxation, socializing and time to reflect on the training or match [7]. Not all of these aspects are specifically assessed with the POMS and rest-Q. Therefore, it is debatable whether questionnaires such as the POMS and rest-Q sport do adequately assess psychological recovery. However, the perceived benefit could also reflect a placebo effect, whereby individuals believe that the active cool-down is more beneficial than a passive cool-down due to the popularity in society and its proposed benefits. Cook and Beaven [27] for example found a correlation between the perception of the effectiveness of a recovery modality and subsequent performance that was of similar magnitude to the correlation observed between physiological recovery and performance, suggesting that the perception of a recovery modality can also have a major influence on its effects.

### Long-Term Effects of an Active Cool-Down

All studies discussed so far have investigated the acute or short-term (< 1 week) effects of an active cool-down and a passive cool-down. In the following two sections we discuss the long-term effects of an active cool-down on injuries and the adaptive response.

### Injury Prevention

An active cool-down can theoretically reduce the risk of injuries during a subsequent training session, because a better recovery may result in less neuromuscular fatigue (see small, non-significant positive effects in Sect. 4.4) and thereby decrease injury risk. Only a few studies have investigated the effects of an active cool-down on injuries, and this has usually been investigated in combination with stretching and a warm-up. In three prospective cohort studies on runners, regular use of a cool-down did not significantly reduce the incidence of running injuries [120–122]. In another prospective study on runners, a health education intervention program consisting of a warm-up, cool-down, and stretching exercises also did not significantly reduce the incidence of running injuries [123]. However, a potential confounder in this study was that most participants in the control group also already performed these practices of their own volition. Finally, performing a regular cool-down after exercise was also not significantly associated with a reduction in injuries among triathletes [124] or with finishing a marathon versus not finishing a marathon in recreational runners [125]. In contrast with the evidence from the studies above, a study on dance aerobics instructors found a significant association between the duration of the cool-down and the number of injuries. Specifically, the group performing a 15-min cool-down showed a lower injury rate than the 5- and 10-min cool-down groups [126], but no control group was included for comparison. Therefore, a cool-down generally does not affect injury rates, although more research is required to investigate the effects of the type of cool-down, its duration, and the type of sport.

### Long-Term Adaptive Response

Exercise stimulates the release of various biochemical messengers that activate signaling pathways, which in turn regulate molecular gene expression that elicits an adaptive response [100]. Some recovery interventions such as antioxidant supplementation, nonsteroidal anti-inflammatory drugs, and cold-water immersion can influence signaling pathways, thereby attenuating the long-term adaptive response to exercise [100, 127, 128]. For example, several studies have shown that cold-water immersion after each training session reduces blood flow and influences signaling pathways, thereby leading to reduced gains in muscular strength and endurance compared to an active cool-down or passive cool-down [129–133]. Similarly, chronic intake of some antioxidants can also have a harmful effect on mitochondrial biogenesis and performance [100, 127, 134]. Preliminary evidence suggests that an active cool-down consisting of 15 min moderate-intensity jogging does not attenuate the long-term adaptive response in well-trained intermittent sport athletes [135]. Interestingly, the group that regularly performed an active-cool down after training even obtained a higher anaerobic lactate threshold after 4 weeks of training compared with the passive cool-down group. This could be related to the extra training volume completed during an active cool-down. However, conflicting evidence for the attenuating effects of other recovery modalities such as cold-water immersion has been reported [136], and more research investigating the effects of an active cool-down on the long-term adaptive response with other exercise modalities (e.g., following strength training and using swimming or cycling during the active cool-down) and populations (e.g., untrained individuals, elderly) is therefore required.

## Combination with Other Recovery Interventions

This review has focused on the effects of an active cool-down consisting of low-intensity exercises such as cycling or running on measures of sports performance, psychophysiological recovery, injuries, and the long-term adaptive response. However, most individuals usually perform a combination of recovery interventions, and this combination may have different effects than an active cool-down in isolation. Two recovery interventions that are frequently performed in combination with an active cool-down are stretching and, more recently, foam rolling. The effects of these cool-down interventions are briefly discussed in the following sections.

### Static Stretching

Stretching—especially static stretching—is frequently incorporated in an (active) cool-down [15, 28, 29, 42] (Table 2). For example, a study among recreational marathon runners reported that 64% of the runners performed stretching after training [122]. Another survey on elite adolescent athletes found that 23% of the Asian and 68% of the UK athletes used stretching after a training session [91]. Finally, a survey among collegiate athletic trainers in the USA found that 61% recommended static stretching to be included as a recovery method after exercise [1]. Surveys among coaches from other sports report similar results [2, 3, 5, 137].

Stretching is usually performed to reduce muscle soreness and increase range of motion. Many practitioners also believe that stretching reduces the risk of injuries and improves performance [1, 3–5]. Contrary to common belief, however, static stretching performed either before or after exercise does not reduce muscle soreness [41, 138]. Although stretching can reduce muscle stiffness (when performed as constant-torque stretching [139]) and increase the range of motion [67], these effects are also not always in the athlete’s interest. Long-distance runners with a better running economy are (for example) actually less flexible, and increasing flexibility can potentially negatively affect running economy [72, 140]. Finally, although static stretching may have some effects on strain injuries [141], an increasing body of research suggests that it has little to no effect on the prevention of degenerative injuries [140]. Therefore, although stretching is historically a widely practiced cool-down activity, it may not necessarily aid recovery from exercise.

### Foam Rolling

Foam rolling has more recently also been incorporated in many cool-downs, although to a lesser extent than stretching. A small proportion (4%) of Asian and moderate proportion (38%) of UK elite adolescent athletes report using foam rolling after training [91]. Foam rolling is frequently performed to reduce muscle soreness and to attenuate the effects of exercise on the reduced range of motion. Indeed, foam rolling performed after exercise has been found to reduce delayed onset of muscle soreness, increase range of motion, and enhance sports performance during the next day [142, 143]. For example, MacDonald et al. [142] found that the foam rolling group demonstrated less muscle soreness and better dynamic (but not passive) range of motion of the hamstrings and vertical jump performance. However, foam rolling also reduced evoked contractile properties during the next day. Similarly, Rey and co-workers [144] reported that 20 min of foam rolling following a soccer practice improved agility performance, the perception of recovery and reduced muscle soreness in professional soccer players. However, foam rolling did not significantly improve sit-and-reach performance or 5- and 10-m sprint performance. Therefore, foam rolling may facilitate recovery from exercise, but more research is needed.

## Conclusions and Practical Applications

**Fig. 2:**
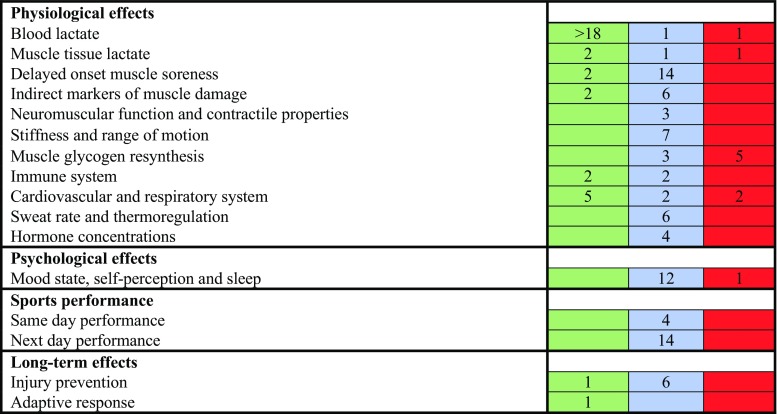
Evidence heatmap showing the effects of an active cool-down on markers of psychophysiological recovery, sports performance, and long-term effects. Numbers represent the number of studies demonstrating a significant benefit (green), no significant difference or an inconclusive effect (blue), or significant harm (red) of an active cool-down on the variable of interest compared to a passive cool-down

Although there are many proposed benefits of an active cool-down compared with a passive cool-down (Fig. 1), this review shows that only a few of these benefits are supported by research (Fig. 2). Most importantly, we have provided evidence that an active cool-down generally does not improve and may even negatively affect performance later during the same day when the time between successive training sessions or competitions is > 4 h. Similarly, an active cool-down has likely no substantial effects on next-day(s) sports performance, but can potentially enhance next-day(s) performance in some individuals (Table 2). With regard to the long-term effects, a cool-down does likely not prevent injuries, and preliminary evidence suggests that an active cool-down after every training sessions does not attenuate and may even enhance the long-term adaptive response.

Several psychophysiological mechanisms are believed to underlie the potential beneficial effects of an active cool-down. This review shows that an active cool-down does generally lead to a faster removal of lactate in blood, but the practical relevance of this findings is questionable, especially because lactate is not necessarily removed faster from muscle tissue and because lactate may not be the cause of metabolic acidosis. Furthermore, an active cool-down can partially prevent the depression of circulating immune cells counts after exercise. However, it is unknown whether this also leads to fewer infections and illnesses. An active cool-down can also result in a faster recovery of the cardiovascular and respiratory system after exercise, but it remains unknown whether this leads to a reduction in the number of post-exercise syncopes and cardiovascular complications. In contrast, an active cool-down generally does not significantly reduce delayed-onset muscle soreness or improve the recovery of indirect markers of muscle damage. It also does not significantly alter the recovery of the neuromuscular and contractile properties, improve range of motion, or attenuate musculotendinous stiffness following exercise, and may even interfere with glycogen resynthesis. Furthermore, an active cool-down does generally not significantly facilitate the recovery of hormonal concentrations, and it also does not affect measures of psychophysiological recovery. However, most individuals nevertheless perceive an active cool-down as more beneficial than a passive cool-down. The effectiveness of an active cool-down may differ depending on the individual preferences and beliefs; recovery interventions should therefore be individualized [28, 30]. Some athletes may benefit more from an active cool-down, whereas others may prefer to perform no cool-down at all.

The mode, intensity, and duration of a cool-down and activity preceding the cool-down will likely influence the effectiveness of the cool-down on recovery and these effects may also differ between individuals. It is therefore difficult to recommend one optimal active cool-down protocol for all individuals in all situations. Some general guidelines can, however, be provided. An active cool-down should: (1) involve dynamic activities performed at a low to moderate metabolic intensity to increase blood flow, but prevent development of substantial additional fatigue; (2) involve low to moderate mechanical impact to prevent the development of (additional) muscular damage and delayed-onset muscle soreness; (3) be shorter than approximately 30 min to prevent substantial interference with glycogen resynthesis; and (4) involve exercise that is preferred by the individual athlete. Some evidence also suggests that an active cool-down should involve the same muscles as used during the preceding activity [145].

More research is required to investigate the differences between different active cool-down interventions (e.g., land-based vs. water-based active cool-downs), the effects of different exercise protocols that precede the cool-down, and the effect of active cool-downs in various populations (e.g., elderly). It is also important to consider that most studies have investigated the effects on untrained or recreationally trained individuals, because the detrimental effects of training are easier to induce (to show greater effects of recovery interventions). These findings may not necessarily transfer to better trained athletes. Finally, several studies have used protocols that are rarely used in daily practice and more research is required on practical active cool-downs and the effects of active cool-downs on endurance performance.
